# RAD-Seq derived markers flank the shell colour and banding loci of the *Cepaea nemoralis* supergene

**DOI:** 10.1111/mec.12262

**Published:** 2013-03-15

**Authors:** Paul M Richards, M Maureen Liu, Natalie Lowe, John W Davey, Mark L Blaxter, Angus Davison

**Affiliations:** * Centre for Genetics and Genomics, School of Biology, University of NottinghamNottingham, NG7 2RD, UK; † Institute of Evolutionary Biology, School of Biological Sciences, University of EdinburghEdinburgh, EH9 3JT, UK; ‡ The GenePool Genomics Facility, School of Biological Sciences, University of EdinburghEdinburgh, EH9 3JT, UK

**Keywords:** colour polymorphism, *Heliconius*, RAD-Seq, restriction site–associated DNA sequencing, snail, supergene

## Abstract

Studies on the classic shell colour and banding polymorphism of the land snail *Cepaea* played a crucial role in establishing the importance of natural selection in maintaining morphological variation. *Cepaea* is also a pre-eminent model for ecological genetics because the outward colour and banding phenotype is entirely genetically determined, primarily by a ‘supergene’ of at least five loci. Unfortunately, progress in understanding the evolution and maintenance of the *Cepaea* polymorphism stalled, partly because of a lack of genetic markers. With a view to re-establish *Cepaea* as a prominent model of molecular ecology, we made six laboratory crosses of *Cepaea nemoralis*, five of which segregated for shell ground colour (*C*) and the presence or absence of bands (*B*). First, scoring of colour and banding in 323 individuals found no recombination between the *C* and *B* loci of the supergene. Second, using restriction site–associated DNA sequencing (RAD-Seq) of two parents and 22 offspring, we identified 44 anonymous markers putatively linked to the colour (*C*) and banding (*B*) loci. The genotype of eleven of the most promising RAD-Seq markers was independently validated in the same 22 offspring, then up to a further 146 offspring were genotyped. The closest RAD-Seq markers scored are within ∼0.6 centimorgan (cM) of the *C-B* supergene linkage group, with the combined loci together forming a 35.8 cM linkage map of markers that flank both sides of the *Cepaea C-B* supergene.

## Introduction

**Fig. 1:**
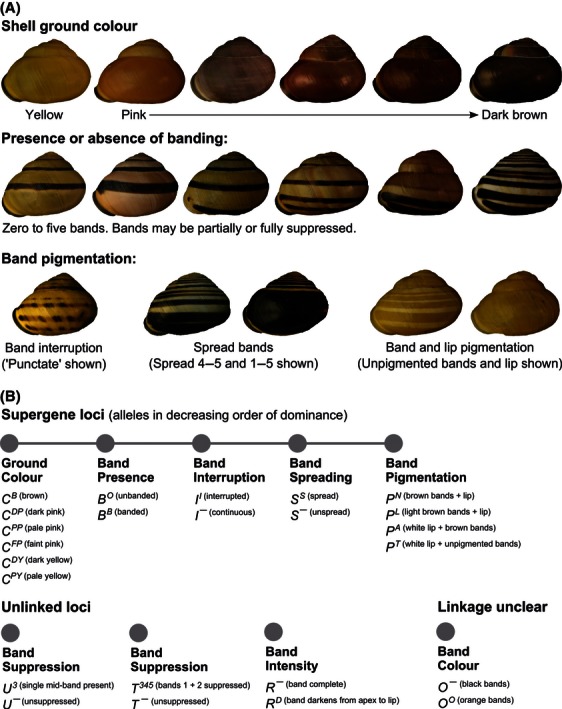
(A) Shell polymorphism in *Cepaea nemoralis*. There is considerable phenotypic variation, and also convergence, in that the same phenotype may be reached by different routes, for example brown- and spread-banded morphs are both very dark. Precise scoring of some phenotypes can be challenging, especially distinguishing some browns and dark pinks. (B) Genetics of the shell polymorphism. Of the nine loci known to control the polymorphism, at least five are tightly linked as a supergene. Physical order is unclear, although ground colour and band presence form the tightest linkage group. The supergene is also found in the sister taxon *Cepaea hortensis*, with simpler versions in *Cepaea vindobonensis* and *Cepaea sylvatica*. Figure 1B is adapted from Jones *et al*. (1977) and Murray (1975).

It is over sixty years since Cain and Sheppard published the first of their seminal papers on adaptive variation in the shell colour and banding patterns of the land snail *Cepaea nemoralis* (Cain & Sheppard 1950, 1952, 1954). Classic work on the *Cepaea* polymorphism contributed to the establishment of the field of ecological genetics and has provided (Jones *et al*. 1977; Clarke *et al*. 1978) and continues to provide (Silvertown *et al*. 2011; Cameron & Cook 2012) compelling evidence for the fundamental role of natural selection in promoting and maintaining variation in natural populations. However, in contrast to other classic ecological genetic systems such as wing pattern variation in mimetic butterflies (Joron *et al*. 2011; Heliconius Genome Consortium 2012), nothing is known about the molecular genetics of the *Cepaea* polymorphism, despite the important mechanistic insights this could provide into how ecology and genetics interact to shape adaptive variation. The *Cepaea* polymorphism (Fig. 1; see Cook (1998), Clarke *et al*. (1978) and Jones *et al*. (1977) for reviews) is defined by extensive phenotypic variation for shell ground colour, the presence of zero to five bands and the pigmentation patterns of these bands (Fig. 1A). Classical genetic studies (Murray 1975; Jones *et al*. 1977 and references therein) have identified nine loci that control this variation, at least five of which are tightly linked together on the same chromosome and so are inherited together as a ‘supergene’ (Fig. 1B). These loci interact epistatically with one another (e.g. the band presence locus with the band pigmentation, spreading and interruption loci) and with the unlinked loci (e.g. suppression of specific bands). Empirical estimates of the frequency of recombination within the supergene, although limited, suggest that shell ground colour (*C*), band presence (*B*) and band interruption (*I*) are in a very tight linkage group, with recombination frequencies typically towards the lower end of ∼0–2% (Cain *et al*. 1960; Cook & King 1966; Cook 1967). Spread banding (S) and band pigmentation (*P*) may be more loosely linked to the *C*-*B* linkage group, with respective recombination frequency estimates of ∼3–4% (Cain *et al*. 1960; Cook 1967) and ∼4–15% (Cook 1967) reported. Certainly, additional estimates of linkage within the supergene would be beneficial.

A variety of processes may be involved in both the original evolution and maintenance of the polymorphism (Jones *et al*. 1977; Clarke *et al*. 1978; Cook 1998). Correlations between colour and banding phenotypes and selective factors, such as climate and predation, indicate a strong directional selection component (e.g. yellow snails are often more common in open grassland habitats, where they are better camouflaged and less liable to overheat than pink or brown snails). The observation that coadapted colour and banding alleles are often found in strong linkage disequilibrium with one another (e.g. yellow-banded in variegated hedgerow and grassland habitats) is consistent with the hypothesis that the supergene may have evolved as a means to prevent these adaptive allelic combinations from being broken up by recombination (Cain & Sheppard 1954; Cain *et al*. 1960). Indeed, given *C. nemoralis* has a haploid chromosome number of 22, the probability of finding five linked loci out of eight (excluding locus *O*, which may or may not be linked) is low, around 2 × 10^−4^. The question of how supergenes evolve and subsequently remain protected from recombination is a longstanding one in population genetics. The problem is certainly more complicated than gene duplication, because typically the linked loci seem to have diverse biochemical and developmental roles (Jones *et al*. 1977; Barrett *et al*. 2000; Nijhout 2003). A further key problem is that bringing coadapted loci together in the genome will also generate irrelevant or harmful allelic combinations. Theoretically, it has been proposed that loci could be brought together as supergenes by chromosomal rearrangements (Sheppard 1953; Charlesworth & Charlesworth 1975; Kirkpatrick & Barton 2006), or a ‘sieve’ mechanism, whereby linkage is tightened between loci that are already located near one another (Turner 1977; D'Ennequin *et al*. 1999). Interestingly, recent molecular evidence suggests a role for both mechanisms in the evolution of a butterfly supergene (Joron *et al*. 2011; Jones *et al*. 2012). Certainly, empirical molecular evidence from *Cepaea* could shed further light on this problem. While directional selection has undoubtedly played an important role in the evolution of the *Cepaea* polymorphism, the situation is complex because balancing selection (e.g. predation-driven apostatic selection) also acts on the polymorphism, such that rare recombinants may be sometimes favoured (Allen 1988; Cameron & Cook 2012). The mechanisms that continually maintain this variation remain difficult to disentangle (Jones *et al*. 1977; Cook 1998). Arguably, however, the evolution of the Cepaea polymorphism, of which the supergene is an inherent component, centres on the tension between balancing selection favouring the novel phenotypes created by rare recombination events within the supergene and directional selection favouring the suppression of recombination and maintenance of coadapted allele combinations. To identify and characterize the supergene and better understand the evolution of the polymorphism will require a set of linked molecular markers. Combining genetic knowledge with the wealth of historical and contemporary population genetic data available online as part of the citizen science project, the Evolution Megalab (http://www.evolutionmegalab.org/) (Silvertown *et al*. 2011; Cameron & Cook 2012), would greatly facilitate new angles of research. Until recently, mapping adaptive loci in *Cepaea*, and other nonmodel organisms, has been inhibited by technological and cost constraints. However, a number of recently developed, rapid and cost-effective genotyping-by-sequencing methods, including restriction site–associated DNA sequencing (RAD-Seq), now meet the demands for *de novo* mapping in nonmodel organisms (Baird *et al*. 2008; Davey & Blaxter 2010; Amores *et al*. 2011; Baxter *et al*. 2011; Davey *et al*. 2011, 2013; Rowe *et al*. 2011). Consequently, with an ultimate view to identify and dissect the individual components of the *Cepaea* supergene, and to understand their evolution, we used RAD-Seq to construct a local map of markers linked to the shell ground colour (*C*) and banding presence (*B*) loci. We also present new data on the frequency of recombination between these two tightly linked loci, based on our own crosses. The map and the resource that are the segregating offspring should form the foundation of future work to re-establish *Cepaea* as a contemporary model of molecular ecology.

## Methods

### The culture of Cepaea

Snails were fed a hydrated grass pellet, oat and chalk mix, supplemented with lettuce. Generally, large juvenile virgin snails were raised to adulthood in isolation and then introduced to a partner. Pairs of snails were then kept in tanks with ∼4 cm soil until egg laying began. As *Cepaea nemoralis* is a simultaneous hermaphrodite, offspring from both parents were used, except for crosses C100 × C101 and C108 × C109, where a nonvirgin snail (C100 and C108 respectively) was removed after mating. Egg batches were isolated, and the offspring reared to adulthood under the same feeding regime, with the time from egg to adult being ∼6 months. Mature offspring and parents were then frozen at −20 °C. The shell ground colour (*C*) and presence or absence of bands (*B*) phenotypes of all snails were then scored. Segregation, including phase, of the colour and band presence loci was inferred from the parental and offspring phenotypes, where shell colour is pink (*C*^*P*^*C*^*P*^ or *C*^*P*^*C*^*y*^) or yellow (*C*^*y*^*C*^*y*^ homozygous recessive) and bands absent (*B*^*O*^*B*^*O*^ or *B*^*O*^*B*^*b*^) or present (*B*^*b*^*B*^*b*^ homozygous recessive). For each cross (Table 1), Mendelian segregation ratios were tested using chi-square goodness-of-fit tests.

**Table 1:** Summary of *Cepaea nemoralis* crosses. Cross 1 was used for linkage mapping; crosses 1 and 6 were used to validate RAD-Seq-derived linked markers; crosses 1–5 were used to estimate frequency of recombination between the ground colour (*C*) and band presence (*B*) supergene loci. All sites are UK unless otherwise stated. Key to phenotypes: Pink-unbanded 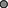; pink-banded 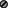; yellow-unbanded 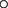; yellow-banded 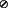. Note: some of the snails used are derived from previous laboratory crosses, so it was not possible to infer the geographic origin of each one of the pair of chromosomes that contain the supergene. Shell colour and banding phenotype data for all individuals from the six crosses are archived in DRYAD under doi:10.5061/dryad.560r4

	Parental chromosomes				F1 offspring genotypes				
			
Cross	Parent	Genotype		Chromosome origin	*C*^*P*^ *B*^*O*^ *C*^*y*^ *B*^*b*^ 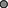	*C*^*P*^ *B*^*b*^ *C*^*y*^ *B*^*b*^ 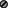	*C*^*y*^ *B*^*O*^ *C*^*y*^ *B*^*b*^ 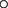	*C*^*y*^ *B*^*b*^ *C*^*y*^ *B*^*b*^ 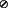	Total *N*
Test cross repulsion					0	156	133	0	289
1	C100	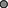	*C*^*P*^ *B*^*b*^ *C*^*y*^ *B*^*O*^	Wye Valley Wye Valley	0	56	47	0	103
	C101	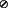	*C*^*y*^ *B*^*b*^ *C*^*y*^ *B*^*b*^	Marlborough Downs Slieve Carron, Ireland					
2	C108	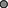	*C*^*P*^ *B*^*b*^ *C*^*y*^ *B*^*O*^	San Roque, Spain San Roque, Spain	0	27	23	0	50
	C109	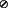	*C*^*y*^ *B*^*b*^ *C*^*y*^ *B*^*b*^	Marlborough Downs Marlborough Downs					
3	C110	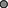	*C*^*P*^ *B*^*b*^ *C*^*y*^ *B*^*O*^	Nottingham Nottingham	0	17	10	0	27
	C111	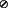	*C*^*y*^ *B*^*b*^ *C*^*y*^ *B*^*b*^	Esles, Spain Esles, Spain					
4	C112	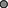	*C*^*P*^ *B*^*b*^ *C*^*y*^ *B*^*O*^	Nottingham Nottingham	0	56	53	0	109
	C113	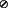	*C*^*y*^ *B*^*b*^ *C*^*y*^ *B*^*b*^	Esles, Spain Esles, Spain					
Test cross coupling									
5	C114	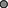	*C*^*P*^ *B*^*O*^ *C*^*y*^ *B*^*b*^	San Roque, Spain San Roque, Spain	18	0	0	16	34
	C115	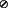	*C*^*y*^ *B*^*b*^ *C*^*y*^ *B*^*b*^	Esles, Spain Esles, Spain					
Segregating for colour (*C*) only									
6	C118	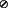	*C*^*y*^ *B*^*b*^ *C*^*y*^ *B*^*b*^	Marlborough Downs Origin unknown	n/a	37	n/a	38	75
	C119*	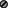	*C*^*P*^ *B*^*b*^ *C*^*y*^ *B*^*b*^	Wye Valley Origin unknown					

* C119 is an F1 from cross C100 × C101. Therefore, the unknown chromosome could originate from the Marlborough Downs or Slieve Carron.

Crosses were generated for three linked purposes: (i) to construct RAD-Seq libraries and generate candidate supergene-linked loci; (ii) to provide further offspring to validate candidate RAD-Seq loci as being linked or not; and (iii) to assess the frequency of recombination within the supergene.

### DNA extractions

Genomic DNA was extracted from frozen snail foot tissue as follows. In brief, slices of snail tissue were incubated at 65 °C in extraction solution (3% CTAB, 100 mm Tris-HCl, pH 7.5, 25 mm EDTA, pH 8, 2 m NaCl) with 0.2 mg/mL proteinase K and 80 μg/mL RNase. Upon lysis, a chloroform extraction was performed, then three volumes of CTAB dilution solution added (1% CTAB, 50 mm Tris-HCl, pH 7.5, 10 mm EDTA, pH 8). Samples were mixed until a precipitate appeared, then the supernatant removed. The pellet was washed twice in 0.4 m NaCl in TE (0.4 m NaCl, 10 mm Tris-HCl, pH 7.5, 1 mm EDTA, pH 8), redissolved in 1.42 m NaCl in TE (1.42 m NaCl, 10 mm Tris-HCl, pH 7.5, 1 mm EDTA, pH 8), then precipitated in ethanol. For RAD library construction, precipitated DNA was spooled out, then washed in 70% ethanol; otherwise, centrifugation was used.

### RAD-Seq library construction

**Fig. 2:**
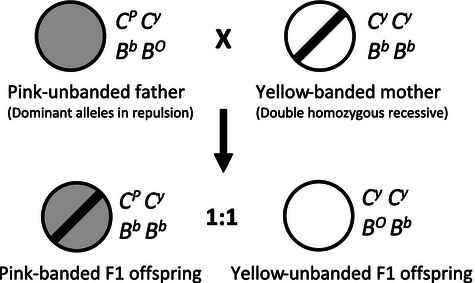
Schematic drawing of the linkage mapping cross (C100 × C101; Table 1). The cross-segregates for the ground colour (*C*) and banding presence (*B*) supergene loci. Pink *C*^*P*^ is dominant to yellow *C*^*y*^, and unbanded *B*^*O*^ is dominant to banded *B*^*b*^. No supergene recombinant offspring were observed out of 103 individuals.

For a single cross (C100 × C101; Table 1; Fig. 2), Restriction site–associated DNA sequencing libraries were constructed as in Baird *et al*. (2008), but with some minor modifications. Briefly, genomic DNA from each individual was separately digested with *Sbf*I and then ligated to a P1 adapter containing a five-base barcode unique to each individual, with a minimum of two-base differences between each barcode. Samples were then pooled and sheared to ∼400 bp fragments on a Covaris S2 sonicator. The fraction of 300–600 bp sheared DNA fragments was size selected from an agarose gel and ligated to a P2 paired-end adapter with the following sequences: P2 top oligo 5′-/5Phos/CTCAGGCATCACTCGATTCCTCCGAGAACAA-3′; P2 bottom oligo 5′-CAAGCAGAAGACGGCATACGACGGAGGAATCGAGTGATGCCTGAG*T-3′, where * denotes a phosphorothioate bond; both oligos of the P1 adapters were also modified with a phosphorothioate bond at the same position. The adapter-ligated DNA was subject to 20 cycles of PCR enrichment followed by gel purification of the 400- to 700-bp fraction of PCR product. Each library was paired-end sequenced on an Illumina GAIIx flow cell, producing 101 base reads.

### Generation of RAD loci

Restriction site–associated DNA sequence analysis was performed using radtools version 1.2.4 (http://www.RAD-Seq.info) as described in Baxter *et al*. (2011). Briefly, for each library, the raw reads were separated into pools, according to the unique five-base barcode assigned to each individual; reads were discarded where sequence quality was not sufficient to recover the barcode or restriction site. Barcodes were then trimmed from the reads to leave 96 base sequences. For each parent, the pooled reads from each library were then concatenated, ready for analysis. For each individual, first read data were clustered into candidate RAD loci comprising one or more candidate RAD alleles; a default clustering distance of up to five bases was allowed between alleles that were grouped together as a single RAD locus, allowing for diversity between the alleles. Only bases with quality scores >20 were used to assess similarity during clustering, and alleles with a read depth <2 were discarded. To account for PCR duplication bias, read counts per allele, per individual, were converted into estimated counts of the actual number of DNA fragments in the original sample (fragment counts); where the associated paired-end reads were duplicated, first reads were collapsed into a single unique sequence. RAD loci appearing in only a single individual were then discarded and the remaining loci merged across individuals, allowing for up to a 3-base mismatch. Merged RAD loci exhibiting >4 alleles were then discarded, because only a maximum of four alleles can segregate for a given locus; loci with >4 alleles are likely to be false positives, resulting from erroneously clustered loci such as repeat regions. The resulting data set of putative RAD loci was used to search for candidate loci linked to the supergene. Read counts and coverage for each individual are given in Table 2.

**Table 2:** Shell colour and banding genotypes, number of Illumina reads sequenced per individual and allelic coverage of the final candidate restriction site–associated DNA (RAD) loci data set (loci found in >1 individual; maximum of four alleles). Twenty-six individuals were multiplexed across three RAD libraries, with each library sequenced on an Illumina GAIIx flow cell (library 2 was run twice and the data concatenated). Illumina read count excludes reads lacking a recognizable barcode or *Sbf*I restriction site. Mean coverage per individual was calculated from all the alleles present in a given individual. The individual means were then weighted by allele count prior to estimating the mean coverage across all individuals. Fragment counts closely represent the true number of DNA fragments in the original sample, after PCR duplicates are discounted

				Final candidate RAD loci data set		
				
Individual	Genotype	Library	Reads	RAD alleles	Coverage (fragments)	SD
Mother yellow-banded	*C*^*y*^*C*^*y*^ *B*^*b*^*B*^*b*^	1	1379099			
		2	693002			
		3	1451806			
		Total	3523907	46612	3.6	3.0
Father pink-unbanded	*C*^*P*^*C*^*y*^ *B*^*b*^*B*^*O*^	1	3793946			
		2	989760			
		3	1418850			
		Total	6202556	47962	3.4	2.6
F1 pink-banded 1	*C*^*P*^*C*^*y*^ *B*^*b*^*B*^*b*^	1	2160005	38999	3.9	3.1
F1 pink-banded 2	*C*^*P*^*C*^*y*^ *B*^*b*^*B*^*b*^	1	3076533	43344	5.2	4.0
F1 pink-banded 3	*C*^*P*^*C*^*y*^ *B*^*b*^*B*^*b*^	1	2966502	43456	4.9	3.9
F1 pink-banded 4	*C*^*P*^*C*^*y*^ *B*^*b*^*B*^*b*^	1	3974551	46018	6.2	4.7
F1 pink-banded 5	*C*^*P*^*C*^*y*^ *B*^*b*^*B*^*b*^	2	1183558	42745	8.0	5.7
F1 pink-banded 6	*C*^*P*^*C*^*y*^ *B*^*b*^*B*^*b*^	2	1370071	45371	8.6	6.5
F1 pink-banded 7	*C*^*P*^*C*^*y*^ *B*^*b*^*B*^*b*^	2	1059479	41983	7.3	5.3
F1 pink-banded 8	*C*^*P*^*C*^*y*^ *B*^*b*^*B*^*b*^	2	1129291	41980	7.6	5.5
F1 pink-banded 9	*C*^*P*^*C*^*y*^ *B*^*b*^*B*^*b*^	3	2422586	44488	5.2	3.9
F1 pink-banded 10	*C*^*P*^*C*^*y*^ *B*^*b*^*B*^*b*^	3	2208664	42599	4.7	3.6
F1 pink-banded 11	*C*^*P*^*C*^*y*^ *B*^*b*^*B*^*b*^	3	3177898	50240	5.7	4.6
F1 pink-banded 12	*C*^*P*^*C*^*y*^ *B*^*b*^*B*^*b*^	3	3271227	45173	6.4	4.9
F1 yellow-unbanded 1	*C*^*y*^*C*^*y*^ *B*^*O*^*B*^*b*^	1	3314935	43942	5.2	4.0
F1 yellow-unbanded 2	*C*^*y*^*C*^*y*^ *B*^*O*^*B*^*b*^	1	3088532	43470	5.1	3.9
F1 yellow-unbanded 3	*C*^*y*^*C*^*y*^ *B*^*O*^*B*^*b*^	1	2870994	40252	4.9	3.8
F1 yellow-unbanded 4	*C*^*y*^*C*^*y*^ *B*^*O*^*B*^*b*^	1	2781767	41671	4.7	3.7
F1 yellow-unbanded 5	*C*^*y*^*C*^*y*^ *B*^*O*^*B*^*b*^	2	2022132	44876	12.5	8.9
F1 yellow-unbanded 6	*C*^*y*^*C*^*y*^ *B*^*O*^*B*^*b*^	2	925077	41203	6.4	4.6
F1 yellow-unbanded 7	*C*^*y*^*C*^*y*^ *B*^*O*^*B*^*b*^	2	1167696	42109	7.9	5.7
F1 yellow-unbanded 8	*C*^*y*^*C*^*y*^ *B*^*O*^*B*^*b*^	2	1366958	43288	8.8	6.4
F1 yellow-unbanded 9	*C*^*y*^*C*^*y*^ *B*^*O*^*B*^*b*^	3	2145951	44009	4.4	3.6
F1 yellow-unbanded 10*	*C*^*y*^*C*^*y*^ *B*^*O*^*B*^*b*^	3	1244963	37248	3.1	2.4
F1 yellow-unbanded 11*	*C*^*y*^*C*^*y*^ *B*^*O*^*B*^*b*^	3	953740	32276	2.7	2.1
F1 yellow-unbanded 12	*C*^*y*^*C*^*y*^ *B*^*O*^*B*^*b*^	3	2336153	44054	4.8	3.7
All individuals (*n* = 26)		Mean	2064858	43053	6.6 (weighted)	
		SD	950895	3389	2.5 (weighted)	

* Data for two individuals were excluded prior to searching for putative loci linked to *C-B*, due to them having lower allelic coverage.

### Identification of candidate-linked RAD loci

A combination of Excel and Unix commands were used to search for RAD loci that exhibited segregation patterns consistent with linkage to the supergene. Data from two individuals were excluded at this stage due to them having substantially lower fragment counts than the other individuals (Table 2). Details of the candidate segregation patterns found are given in Table 1 and Table S1, Supporting information. Searches were conducted by first identifying all RAD loci where one of the alleles exhibited a presence/absence pattern across the parents and offspring, which was consistent with linkage in phase with the dominant colour allele (pink *C*^*P*^) or the dominant banding allele (absent *B*^*O*^) (Fig. 2). A threshold of allelic dropout in two offspring was used to account for insufficient sequencing depth. Two putative recombinant offspring were also allowed, to account for the possibility that a recombination event could have occurred close to the colour or banding loci in one or more offspring. Then, for each of the RAD loci in this subset, the presence/absence patterns of the alternative alleles were assessed to see whether they exhibited a segregation pattern consistent with linkage in phase. Again, in these alternative alleles, dropouts were allowed in some individuals (maximum of five where an allele is expected to present in all 24 individuals and a maximum of two where an allele is expected to present in 14, or less, individuals).

One problem was that radtools is not able to deal with indels in the tag sequence. Another potential problem is in identifying pairs of tags where the polymorphism between alleles exceeds that defined by the clustering parameters. To find these missing loci, singleton RAD loci that had been identified in the initial search were compared against all RAD alleles using blastn. Significant matches with ≥90% identities were then manually aligned to see whether the alleles grouped into a locus that cosegregated with either colour or banding (using the same dropout thresholds). RAD loci meeting the aforementioned criteria were considered as potential candidate-linked markers for further investigation.

### Marker development strategy

We aimed to prioritize the development of those candidate markers suspected to be most closely linked to the *C-B* supergene. However, we also developed some lesser linked markers to aid in orientating the closest linked markers with respect to *C-B* and to facilitate rapid validation of other candidate markers (see below). Consequently, of 44 markers (see Results), 11 (Cne_RAD01 to Cne_RAD11) were developed through to an assay. For the remaining 33 candidate markers, we compared the existing RAD-Seq data for each marker to the segregation of the first 11 developed markers. By comparison with individual snails known to have received a recombinant chromosome (between the *C-B* supergene and a RAD-Seq marker), it was possible to infer likely orientation and linkage of new markers relative to the *C-B* supergene. A four step procedure was used—(i) the segregation patterns of the snails that were used to create the RAD-Seq library were inspected for the eleven developed loci. (ii) The location of any recombination events with respect to the *C-B* supergene was inferred in each snail by comparison with the linkage map data. (iii) By inspection of the RAD-Seq segregation patterns of the remaining 33 loci, it was possible to estimate linkage to the established 11 loci and *C-B*. (iv) PCR primers were designed for the few markers that were suspected as being more closely linked. The locus-specific PCR products were then directly sequenced in the parents, as well as the single offspring individual that was identified as containing a chromosome that is recombinant between the tightest linked map markers and the supergene.

### Validation of RAD-derived markers

Restriction site–associated DNA loci that were identified as being putatively linked to the supergene from the bioinformatic analysis (Table 3) were validated, by developing them into full markers for screening in a larger number of offspring from C100 × C101 and, in some cases, C118 × C119 (C119 being an F1 from C100 × C101; Table 1). Prior to developing these markers, paired-end contigs were assembled for all RAD alleles using velvetoptimiser version 2.1.7, distributed with velvet 1.1.04 (Etter *et al*. 2011; Davey *et al*. 2013). For each candidate RAD marker, the velvetoptimiser-assembled paired-end contigs associated with each allele were manually checked and further minor edits made. From these high-quality contig assemblies, genotyping assays were designed, based either on length polymorphism or on cleaved amplified polymorphic sequence sites (CAPS) (Konieczny & Ausubel 1993; Thiel *et al*. 2004), using standard PCRs, GeneAmp PCR Gold according to manufacturer's instructions and an annealing temperature of either 58 or 60 °C. For length polymorphism assays, the PCR product was visualized on an agarose gel for scoring. For CAPS assays, 7–10 μL of PCR product was digested in a 20 μL reaction with restriction enzyme and buffer and visualized on an agarose gel for scoring (see Table 3 for markers and their corresponding assays). The contig assemblies for the markers used to construct the linkage map are archived in DRYAD under doi:10.5061/dryad.560r4.

**Table 3:** Segregation patterns of assayed candidate-linked restriction site–associated DNA (RAD) markers. The binary segregation patterns are based on those used by radtools, where ‘1’ indicates presence of a RAD allele in an individual and ‘0’ indicates absence. The individuals are ordered: pink-unbanded father, yellow-banded mother, 12x pink-banded offspring, 9x yellow-unbanded offspring. The first two rows show the patterns expected for markers that fully cosegregate for either banding or colour across all individuals. An ‘x’ indicates where a RAD allele did not occur in an individual due to insufficient coverage, although the allele's presence was subsequently confirmed by PCR. All candidate-linked RAD markers and explanations of the expected segregation patterns are given in Table S1, Supporting information

		Segregation pattern			
					
Marker	Allele	PyPPPPPPPPPPPPyyyyyyyyyy ObbbbbbbbbbbbbOOOOOOOOOO	Assay	Fragment sizes (bp)	Primer sequences
Example	Unbanded Banded	100000000000001111111111 111111111111111111111111			
Example	Pink Yellow	101111111111110000000000 111111111111111111111111		Bold numbers denote undigested PCR fragment size	
Cne_RAD01	Unbanded Banded	100000000010011111111111 111111111111111111111111	CAPS *Rsa*I	61, 226, **287** **287**	F 5′GTGAAATTGCTGACCCCTGT R 3′GCTGGAAAATCTCGGATAGG
Cne_RAD02	Unbanded Banded	10000000000000111x111111 11111111111111xxx1111111	CAPS *Ava*II	70, 309, **379** **379**	F 5′GCAGGCACTGTGAATAAGTCAA R 3′CTTATTGACTCGCCCTCGTA
Cne_RAD03	Unbanded Banded	100000010000011111111111 111111111111111111111111	Indel	**211, ˜260** **˜260**	F 5′TCCTGGTAACCCATTTCAGG R 3′CGTGCTGTAATACACATCATCATC
Cne_RAD04	Unbanded Banded	100100000000001111111011 111111111111111111111111	CAPS *Msp*A1I	17, 191, **208** **208**	F 5′TGCAGGGATAGACTCAGCG R 3′TCAAATGAGAATAATGCCAATGA
Cne_RAD05	Pink Yellow	101011111111110000000000 11x11x11xx1111x111111111	CAPS *Alu*I	17, 25, 70, 75, 215, 280 17, 25, 75, 280 (PCR = **397**)	F 5′GGTGGCGACGAGTCTGTATT R 3′TGTCATTGTGCTATTTGTTTCG
Cne_RAD06	Pink Yellow	101111111101100000000000 11x111111x1x111111111111	CAPS *Dde*I	33, 36, 251, 284 36, 284 (PCR = **320**)	F 5′GCCTATCCGTCATTGTTGGT R 3′GTCAAGGCTTGCTTCTTTGG
Cne_RAD07	Pink Yellow	101011111111110000000100 111111111111111111111111	CAPS *Dra*I	21, 192, **221** **221**	F 5′TGCAGGAGGACGATAGTAGC R 3′TGAAGGTTCACCGCAGTAGAT
Cne_RAD08	Unbanded Banded	100000000000001111111111 x11111111111111111111111	CAPS *Hinf*I	44, 222, **300** 44, 222	F 5′GAGGTCAGTATGGCCGAATG R 3′AACACACACAAGCACAAGCA
Cne_RAD09	Unbanded Banded	100000000000001111111111 111111111x1111x111111111	CAPS *BstU*I	**336** 67, 269/260 (length poly.)	F 5′TTTCTCGGAACGACGGAGT R 3′GGTCTCGTCAATGGCACTTT
Cne_RAD10	Unbanded Banded	100000000000001111111111 x1x111111111111111111111	CAPS *Dpn*II	11, 167, **178** **178**	F 5′TTGGTTGGCGTGAtmGAGAGG R 3′GTCTGGGTTAGCTTTCCCGATT
Cne_RAD11*	Pink Yellow	101111111101100000000000 111111111111111111111111	CAPS *Bst*UI	∼200+ ∼190 present ∼200+ ∼190 absent	F 5′AAGAAGCGTCCTTCTGGAAA R 3′CACCTTCCCCATTCTTCAAA

CAPS, cleaved amplified polymorphic sequence sites.

* For marker Cne_RAD11, the polymorphism in the RAD-tag sequence is in phase with banding (see Table S1, Supporting information). However, the SNP used to design the assay, which is derived from the associated paired-end contig assembly, is in phase with colour; this is the segregation pattern shown. The restriction digest produced up to 5 fragments (including an undigested 383-base pair fragment), although only the two fragments shown are diagnostic for linkage to the supergene.

### Linkage map

The validated RAD markers and segregating supergene loci were used to create a linkage map for the C100 × C101 cross, using crimap version 2.5.3 (Green *et al*. 1990). First, two-point recombination frequencies were calculated for each pair of markers to determine that they formed a single linkage group (LOD ≥ 3.0). The relatively small number of markers and individuals meant that the markers could be manually ordered; this assumed fixed order was used in crimap to build a maximum-likelihood linkage map, using Kosambi's mapping function. The reliability of the inferred marker order was confirmed using the ‘flips’ option, comparing the likelihood of the most likely marker order with alternative orders. A schematic of the linkage map was made using mapchart 2.2 (Voorrips 2002).

## Results

### Cepaea crosses and recombination between the supergene loci

Six crosses were generated (398 offspring; Table 1), of which five segregated for both the colour (pink *C*^*P*^ or yellow *C*^*y*^) and banding (presence *B*^*b*^ or absence *B*^*O*^) loci, with the other segregating only for colour. For the former, all crosses significantly deviated from expected Mendelian segregation ratios, as expected if the two loci are tightly linked (combined testcross repulsion populations: *χ*^2^ = 292.7, 3 d.f., *P* ≤ 0.001; single testcross coupling population: *χ*^2^ = 14.2, 3 d.f., *P* ≤ 0.001). Zero recombinants were observed out of 323 individuals. Taking the probability of zero recombinants in *n* = 323 meioses, given the recombination rate (θ) as (1−θ)^*n*^, and a flat prior distribution for θ, the 95% upper confidence limit for θ was calculated from the posterior distributions as 0.5% between the colour (*C*) and banding (*B*) supergene loci.

### Searching for supergene-linked loci using RAD-Seq

Twenty-six *Cepaea nemoralis* snails were genotyped by RAD-Seq, all originating from a single mapping cross, C100 × C101 (Table 1; Fig. 2). This cross comprised 103 offspring that segregated for the colour (*C*) and banding (*B*) supergene loci. The cross was between a nonvirgin heterozygous pink-unbanded (*C*^*P*^*C*^*y*^ *B*^*b*^*B*^*O*^) father and a double homozygous recessive yellow-banded (*C*^*y*^*C*^*y*^ *B*^*b*^*B*^*b*^) mother. The resulting offspring phenotypes were pink-banded (*C*^*P*^*C*^*y*^ *B*^*b*^*B*^*b*^) and yellow-unbanded (*C*^*y*^*C*^*y*^ *B*^*b*^*B*^*O*^), implying zero recombination between the colour-banding element of the supergene. The dominant alleles were in repulsion (i.e. on separate chromosomes) in the heterozygous father and therefore segregated separately from one another in the nonrecombinant offspring (Fig. 2). Conveniently, this meant that RAD markers in phase with banding could be distinguished from markers in phase with the colour. Three RAD-Seq libraries were generated, each comprising the same two parents and eight different offspring. Two libraries were sequenced across one flowcell of an Illumina GAIIx (Table 2; runs 1 and 3), with the other library sequenced on another two flowcells before concatenation (run 2). Across the three libraries, 61.9 million sequences with a recognized barcode and restriction site were generated, giving a mean number of reads per individual of >2 million (SD = 950895; Table 2). These sequences are available via the NCBI SRA database under accession ID: SRA063152. Using radtools, an initial 174 779 candidate alleles were found in more than one individual and clustered into 59 954 loci. After loci with >4 alleles were discarded (erroneously clustered loci, such as repeats), the resulting data set included 57 750 candidate loci, comprising 86 230 alleles, with a weighted mean fragment depth across all individuals of 6.6 (SD = 2.5). The number of candidate RAD loci found corresponds well to a prediction of 52 520, assuming 6.6 Gb diploid genome (average of four helicid snails listed on http://www.genomesize.com/) and a 37% GC content (estimated from *Biomphalaria glabrata*; Adema *et al*. 2006). However, coverage was lower than was aimed for ∼10×, due to a high proportion of presumed PCR duplicates—an average of 66.7% (SD = 16.1%) across all individuals (calculated from all putative RAD alleles found in an individual with a read depth >1). Searches for putative RAD markers that exhibited presence/absence patterns consistent with full cosegregation with the supergene were conducted based on the parents and 22 of the original 24 offspring (two were excluded due to lower coverage, which would result in many dropouts; Table 2). Stringent search criteria, not allowing for any loss of alleles in individuals, returned only a single candidate marker (identified by blastn), which was in phase with banding. Less stringent search criteria yielded a total of 17 markers in phase with colour, 23 markers in phase with banding (including the one above), and four markers for which phase with colour or banding was not known, giving 44 potential candidates to develop further (Table S1, Supporting information).

### Validation of RAD-Seq markers and creating the supergene linkage map

**Fig. 3:**
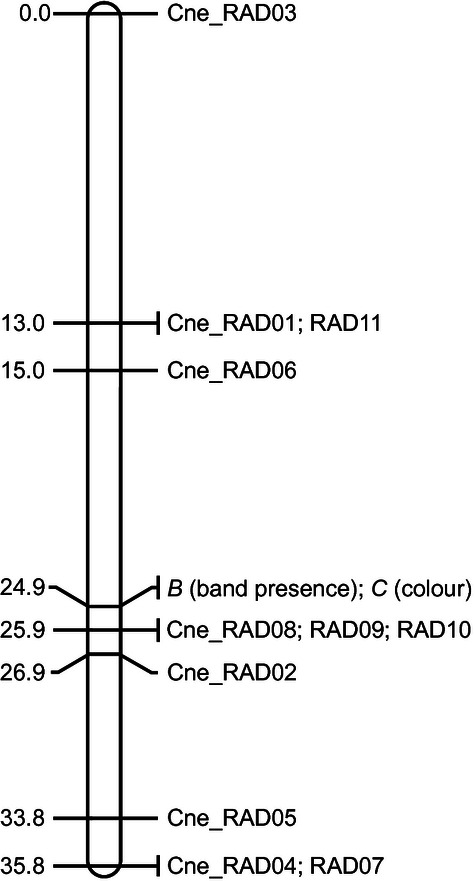
Linkage map of restriction site–associated DNA (RAD) markers flanking the *Cepaea nemoralis* colour (*C*) and banding presence (*B*) supergene loci. The 35.8 cm map was inferred from 11 bi-allelic RAD markers and the phenotypes of the ground colour (*C*) and banding presence (*B*) loci scored in 102 offspring from a single cross (C100 × C101; Table 1). Three markers (Cne_RAD08, Cne_RAD09 and Cne_RAD10) fall within ∼1 cM (1 recombinant in 102) of the *C-B* supergene linkage group. Cne_RAD08 and Cne_RAD10 were also scored in an additional 66 individuals from another cross (C118 × C119; Table 1), placing them ∼0.6 cM (1 recombinant in 168) from *C-B*.

We attempted to validate the 44 loci as being linked or not, primarily prioritizing further development of markers that were suspected to be most closely linked to the *C*-*B* supergene locus (Table S1, Supporting information). This strategy led us to develop full genotyping assays for 11 of the candidate RAD marker loci (Table 3). These loci were genotyped in the parents, the original 22 individuals from the C100 × C101 cross and 80 further offspring from the same cross (one individual not used). This data, in addition to phenotypic scoring of the genotypes of the colour and band presence supergene loci, were then used to construct a local linkage map. The eleven RAD markers and two supergene loci formed a 35.8 cM linkage group (Fig. 3). Three RAD markers (Cne_RAD08, Cne_RAD09 and Cne_RAD10) were located 0.98 cM (1 recombinant in 102 individuals) away from the supergene. Markers Cne_RAD08 and Cne_RAD10 were also scored in 66 individuals from another cross, C118 × C119 (C119 being an F1 from C100 × C101; Table 1). No further recombinants were discovered, putting the total number of recombination events between these loci and the colour and banding loci of the supergene at 1/168 or ∼0.6 cM. The linkage mapping genotypes data set is archived in DRYAD under doi:10.5061/dryad.560r4.

Of the remaining 33 loci, we were most interested to find any that were more closely linked to *C-B* than Cne_RAD08/09/10. Although four loci were identified as being of high priority because the segregation patterns indicated likely strong linkage (including the single locus that showed perfect segregation, mentioned above), we were not able to develop a successful assay for them due to difficulty in alignment, designing primers and PCR. Three were discarded as erroneous or false positive unlinked loci because putative recombinants found in their segregation patterns did not correspond to the RAD offspring validated as recombinants in the linkage map. The remaining 26 loci were identified as being of limited interest at present, because the linkage of each RAD-Seq locus to *C-B* was estimated to be no closer than the existing tightest linked map markers (Cne_RAD08/09/10). Of these 26 loci, 10 were inferred to be orientated on the same side of *C-B* as Cne_RAD08/09/10 and 16 on the opposing side. Full validation details and linkage estimates are given for all 44 markers in Table S1, Supporting information.

## Discussion

We used RAD-Seq to identify the first molecular markers that are closely linked to the *Cepaea* supergene and then derived a local genetic map for this region. The 11 markers assayed thus far form a 35.8 cM linkage group with the closest linked markers falling ∼0.6 cM away from the shell ground colour (*C*) and band presence (*B*) supergene loci. In addition, we found zero recombinants between the colour (*C*) and banding loci (*B*) in 323 individuals, giving an upper limit of 0.5% recombination. This figure is similar to previous estimates of between 0.02% and 2% (Cain *et al*. 1960; Cook & King 1966), consistent with tight physical linkage within the supergene. Together the linkage map and the crosses provide the basis for further research to fine map and, ultimately, to identify the supergene, as well as to understand the evolution and maintenance of the *Cepaea* polymorphism. Development of individual RAD markers was sometimes challenging. First, we found that additional screening was needed during marker development as, with one exception, we did not find RAD loci that fully cosegregated with colour or banding, across all the individuals that were sequenced. The main explanation is insufficient depth of coverage, due to a high proportion of PCR duplicates (Baxter *et al*. 2011; Davey *et al*. 2011; Etter *et al*. 2011), resulting in a relatively high number of allelic dropouts—even for the most closely linked of the eleven RAD markers. A second problem was difficulty in developing suitable assays for some markers, either because of a poor paired-end assembly (perhaps indicating the locus fell within a repeat region), or because of a lack of SNPs in suitable phase (a particular problem for tri-allelic loci). However, these issues were partly negated by the fact that we were focussing on finding a small number of candidate linked loci; this meant it was viable to compare PCR and RAD-Seq segregation data to determine whether absent alleles were real or sequencing artefacts.

### Future fine mapping of the supergene

The genetic map is a preliminary, but important resource for leading the future direction of research on *Cepaea*. Fine mapping of the supergene is an obvious next step, which may be aided by taking a number of different approaches, such as mapping the other loci that comprise the polymorphism, creation of a whole-genome linkage map and association mapping in natural populations. It is worth emphasizing that we have initially considered only the two most easily scored and intensively studied loci of the five *Cepaea* supergene loci (Fig. 1). However, given that observed recombination frequencies between the *C-B* linkage group and the pigmentation (*P*) and spread band (*S*) loci range between 3% and a maximum of 15% (Cain *et al*. 1960; Cook 1967), our closest linked RAD-Seq markers could already fall within the supergene. The creation of additional crosses that segregate for these other supergene loci is underway and will undoubtedly be important for understanding the context of our existing map as well as the structure of the supergene. Development of a whole-genome linkage map will also be of great importance. The low and highly variable sequencing coverage of our current data set, as well as the limited number of included offspring, mean that a genome map was beyond the scope of the current work. However, future work will focus on using a lower number of PCR cycles, to reduce PCR duplicates, achieving high uniform coverage across individuals by synthesizing, and then pooling libraries, based on a few individuals (Baxter *et al*. 2011), and sequencing these to a greater depth using the latest Illumina Hi-Seq technology. Complexity reduction in the RAD libraries, either through the use of a less frequent cutting restriction enzyme or bioinformatically, could also be a route to generating a robust set of markers for a whole-genome map, although this would still need to be coupled with higher coverage than was achieved here. Finally, of critical importance if we are to fine map the individual component loci of the supergene, is to identify recombinants within the supergene. This may be achieved using two complementary methods, first by detecting rare recombinants in further genetic crosses or, alternatively, by linkage disequilibrium or association mapping SNPs from natural populations. The latter method would rely upon the fact that different combinations of colour and banding alleles are frequent in linkage disequilibrium in separate populations, and so distinct populations are likely to represent independent cross-over events within the supergene.

### Evolution of the Cepaea polymorphism: a molecular perspective

While our study represents only the initial steps towards a molecular genetics-led investigation of the *Cepaea* polymorphism, we already envision a number of novel avenues of research. These centre on trying to understand, both at molecular and population levels, the tension between directional selection and balancing selection (in addition to nonselective forces, such as migration), which have driven the evolution and maintenance of the supergene (Jones *et al*. 1977; Clarke *et al*. 1978; Cook 1998). First, by what molecular mechanisms were coadapted colour and banding loci brought together into a supergene, and how have they been subsequently protected from recombination? Moreover, are these loci in tight physical linkage, or do they lie across a larger chromosomal region of suppressed recombination? In the case of the evolution and maintenance of a supergene for mimetic wing pattern variation in the butterfly *Heliconius numata*, a classic example of negative frequency-dependent balancing selection like *Cepaea*, recent targeted and whole-genome sequencing data have implicated both initial tight linkage of the supergene components (Jones *et al*. 2012) and local chromosomal inversions (Joron *et al*. 2011). In comparison, while the *Cepaea* supergene remains anonymous at present, it has been speculated that chromosomal rearrangements are involved; one of the 22 chromosome pairs in *Cepaea nemoralis* is much larger than the others, constituting ∼15–20% of the genome. Certainly, in the first instance, it would be desirable to explore the use of physical mapping, via fluorescent *in situ* hybridization (FISH) (Adema *et al*. 2006), and whole-genome linkage mapping to see whether closely linked RAD-Seq markers map to this large pair of chromosomes. Addressing the population genetics of the *Cepaea* polymorphism using the supergene-linked markers will also be vital for understanding the historical and contemporary evolution of the polymorphism. While past studies, using allozymes (Johnson 1976; Ochman *et al*. 1983), mitochondrial DNA and microsatellites (Davison 2000; Davison & Clarke 2000), have attempted to address the local history of polymorphism, they are limited by the imperfect resolution they provide and the fact that they may share a different history to the supergene. In contrast, RAD markers that are in linkage disequilibrium with the supergene will probably share the same phylogeny as the supergene, so when used in combination with neutral loci, are likely to be more informative for inferring the adaptive history of the polymorphism, both at regional and local scales (Wilding *et al*. 2001; Hines *et al*. 2011). In conjunction with population genetic data, looking at patterns of linkage disequilibrium in the region within and surrounding the supergene may tell us something about the strength and type of selection operating, the length of time variation has been maintained in populations and in turn may shed light on the population processes that have contributed to its maintenance (Charlesworth 2006). For example, sequence diversity within and surrounding the *H. numata* supergene (Joron *et al*. 2011) may be indicative of balancing selection acting within populations having a more important role in maintaining polymorphism than local adaptation and migration between populations (Charlesworth & Charlesworth 2011). Finally, a future line of research will be to use the molecular data generated from *C. nemoralis* to study the degree of synteny with its sister species *Cepaea hortensis*. Given that the polymorphism is phenotypically and genetically (Murray 1975) similar, if not identical, between the two, it is likely that the supergene existed prior to their divergence into separate species, suggesting that we may find trans-specific polymorphism, or else introgression (Jones *et al*. 1977; Heliconius Genome Consortium 2012). Moreover, across the pulmonates as a whole, it is likely that shell polymorphisms and even supergenes have independently evolved multiple times (Clarke *et al*. 1978), raising the question of whether the same loci have been co-opted. High-throughput sequencing has clearly revolutionized what can be achieved in all of the different fields of molecular ecology (Tautz *et al*. 2010), particularly for nonmodel organisms (Ekblom & Galindo 2011). It is understandable that RAD-Seq is rapidly becoming the method of choice for generating ‘reduced representation’ genomic data in nonmodel systems (Rowe *et al*. 2011), be it for genetic mapping (Amores *et al*. 2011; Baxter *et al*. 2011; Gagnaire *et al*. 2013; Hecht *et al*. 2013; Takahashi *et al*. 2013), generating markers for population studies (Emerson *et al*. 2010; Corander *et al*. 2013; Hess *et al*. 2013; Keller *et al*. 2013) or aiding both the assembly and inferences that can be made from whole-genome sequencing (Heliconius Genome Consortium 2012). In our case, we have been able to take a classic polymorphism for which no molecular genetic data existed and rapidly generate a set of linked markers, which will form the foundations of future work to re-establish *Cepaea* as a prominent model of molecular ecology.

## Data accessibility

1. The Illumina 100-bp RAD-tag and paired-end sequences for each individual are available via the NCBI SRA database under accession ID: SRA063152.
2. The RAD-tag sequences and segregation patterns of all 44 RAD loci that were found to putatively cosegregate with shell colour or banding are supplied as online supporting information (Table S1, Supporting information).
3. Assemblies of RAD-tag and associated paired-end sequences for each of the linkage map markers, the data set of genotypes used to construct the linkage map, and the shell colour and banding phenotype data for all individuals from the six crosses (Table 1) are archived in DRYAD under doi:10.5061/dryad.560r4.

## Supporting information

**Table S1** All candidate supergene-linked RAD loci and details of their validation, grouped by the different co-segregation patterns expected for supergene-linked markers.
