# Supplementary material for: RAD-Seq derived markers flank the shell colour and banding loci of the Cepaea nemoralis supergene
Source: Mol Ecol. 2013 Mar 15;22(11):3077–89. doi: 10.1111/mec.12262 (PMC3712483; doi:10.1111/mec.12262)
Supplement: Supplementary file 1 [file mec0022-3077-SD1.docx]

**Table S1**

All candidate supergene-linked RAD loci and details of their validation, grouped by the different co-segregation patterns expected for supergene-linked markers. The search criteria, which allowed for some degree of mismatch to the expected patterns, are described in detail in the methods. The segregation pattern follows that used by RADtools, with presence of an allele in an individual indicated by a ‘1’ and absence indicated by a ‘0’. The order of the individuals in the binary segregation patterns corresponds to the order given in the ‘individual coverage’ column. Also indicated by "BLASTN" are those markers that were identified manually from singleton RAD loci output by RADtools (see the methods section for a detailed explanation). **Type I** candidates (n=11) were developed into full linkage map markers (Table 3 and Figure 3). **Type II** candidates (n=4) exhibited no putative recombinants in their RAD segregation patterns, but due to difficulty in alignment, designing primers and PCR were not developed into Type I markers. Three of these markers have drop outs or may represent one stack of many loci. **Type III** candidates (n=10) were inferred to be orientated on the same side of *C-B* as Cne_RAD08/09/10 Type 1 markers, but at best no more tightly linked to the *C-B* supergene as Cne_RAD08/09/10. These have not have not been developed further at present because they are of limited immediate use. **Type IV** candidates (n=16) are as Type III, except have the opposite orientation relative to the *C-B* supergene. **Type V** candidates (n=3) were not developed because their RAD segregation patterns are either erroneous or they are false-positive unlinked loci. See Methods for full details of the strategy that was used to prioritize the development of loci that were most closely linked to the *C-B* supergene.
